# Collaboration Between a Maxillofacial Surgeon and an Orthodontist in Implementing Comprehensive Rehabilitation Strategies for Newborns with Cleft Lip and Palate

**DOI:** 10.3390/dj14060375

**Published:** 2026-06-17

**Authors:** Olesya Viktorovna Dudnik, Adil Askerovich Mamedov, Andrey Mikhailovich Dybov, Francesco Guido Mangano, Daria Konstantinovna Yudina

**Affiliations:** 1 Department of Orthodontics and Dental Disease Prevention, Sechenov First Moscow State Medical University, 121059 Moscow, Russia; 2 State Budgetary Healthcare Institution «Children’s City Clinical Hospital No. 9 Named After G. N. Speransky», 129329 Moscow, Russia

**Keywords:** unilateral cleft lip and palate, bilateral cleft lip and palate, newborn, orthodontics, mini-implants, surgery, cheiloplasty

## Abstract

**Objectives**: A comprehensive diagnosis and treatment were carried out in 104 newborns, including 48 children diagnosed with unilateral cleft lip and palate (UCLP) and 56 patients diagnosed with bilateral cleft lip and palate (BCLP). The control group consisted of 116 medical records and diagnostic models of jaws from newborns with UCLP/BCLP, which were analysed before and after orthodontic correction using a removable facebow with headgear attachment. **Methods**: All newborns (n = 104) underwent orthodontic correction to reposition of the alveolar process fragments in UCLP and intermaxillary bone in BCLP using mini-implants and elastic traction devices/springs, followed by primary cheiloplasty. Cheiloplasty was performed one month after fragment alignment had been achieved. **Results**: Statistical analysis showed that orthodontic correction using mini-implants and elastic traction was highly effective. In the UCLP group, normalised alignment of alveolar fragments was achieved in 97.9% of cases (*p* = 0.0000000016); in the BCLP group, normalised positioning of the intermaxillary bone was observed in 96.42% (*p* = 0.00000000007). A direct comparison between the treatment and control groups revealed consistent significance across all diastasis measurements, supporting the clinical advantage of fixed orthodontic approaches. **Conclusions**: The clinical data and statistical analysis indicate that the fixed orthodontic appliance combined with mini-implants and elastic traction is effective. This approach normalises alveolar and intermaxillary positioning and provides optimal preoperative conditions for primary cheiloplasty and subsequent uranoplasty. It also shortens rehabilitation duration and leads to stable aesthetic and functional results.

## 1. Introduction

Cleft lip and palate (CLP) represent a significant global healthcare burden, affecting approximately 1 in every 500 to 1000 live births worldwide, underscoring the urgent need for effective therapeutic strategies and highlighting the significant medical and social burden associated with this condition [1,2].

The intricate nature of CLP necessitates the adoption of a comprehensive, multidisciplinary approach to patient management. This complex condition compromises multiple anatomical structures—including the jaws, palate, nose, and lips—resulting in significant functional impairments, including respiration, sucking, swallowing, and speech [3,4]. Moreover, it exerts a profound impact on the psychosocial well-being of both the affected child and their family unit [5,6].

Timely intervention is paramount, particularly given the high plasticity of neonatal tissues, which facilitates more effective correction of deformities. Current treatment protocols emphasise team-based collaboration among neonatologists, paediatricians, maxillofacial surgeons, orthodontists, otolaryngologists, speech therapists, and clinical psychologists [5,6]. This cooperative approach facilitates early identification of associated anomalies, optimisation of treatment planning, and reduction in complication risks.

Despite the availability of modern treatment modalities, significant limitations persist. Removable orthodontic appliances often fall short due to challenges in securing them within edentulous jaws, posing aspiration risks, and requiring complex maintenance regimens [4,7]. These devices frequently fail to generate adequate corrective force, particularly in severe bilateral CLP cases, while also disrupting infant feeding and sleep patterns [8,9].

Recent advancements in CLP management have introduced fixed orthodontic systems incorporating mini-implants and elastic traction mechanisms. These innovations address key limitations of conventional methods by providing stable anchorage and precise control over bone fragment positioning [10,11]. However, a critical gap remains in the development and implementation of effective early intervention strategies capable of optimising treatment outcomes for CLP patients.

The disparity between current treatment options and the imperative for improved outcomes necessitates the development of an optimised interdisciplinary approach. This research proposes the following hypothesis: the implementation of a specialised orthodontic care protocol, grounded in interdisciplinary cooperation and utilising fixed orthodontic appliances with mini-implants and elastic traction during the neonatal period, will significantly enhance treatment outcomes in infants with cleft lip and palate [12,13,14].

This hypothesis is further supported by the need to address unmet clinical demands in modern CLP management and the potential of innovative fixed orthodontic technologies to overcome existing limitations in neonatal care [15,16,17,18]. The proposed approach aims to improve anatomical correction efficiency, enhance functional rehabilitation, facilitate natural feeding patterns, mitigate psychosocial impact, and optimise conditions for subsequent surgical interventions.

Aim—To improve rehabilitation outcomes in newborns with cleft lip and palate through presurgical orthodontic treatment using fixed appliances.

## 2. Materials and Methods

This study was a controlled clinical investigation conducted in compliance with the Russian regulatory framework for biomedical research involving human participants, specifically Federal Law No. 323 of 21 November 2011 on the Fundamentals of Protecting the Health of Citizens in the Russian Federation. In line with ethical and legal requirements for clinical research, written informed consent was obtained from the patients’ parents. The consent form outlined the study’s goals and explained the potential risks and benefits. The study was reviewed and approved by the Ethics Committee of Sechenov University (I. M. Sechenov First Moscow State Medical University) of the Ministry of Health of the Russian Federation.

Prior to the study launch, all participating specialists underwent comprehensive training on the research protocol, including assessment methods and interpretation of predefined criteria. Unified standards and coding systems for result documentation were established to ensure consistency across the board. Throughout the study, reproducibility was rigorously monitored: inter-rater agreement among specialists reached approximately 95%. In addition, regular recalibration sessions were conducted for clinicians to maintain result consistency and alignment over time.

**Scheme 1:**
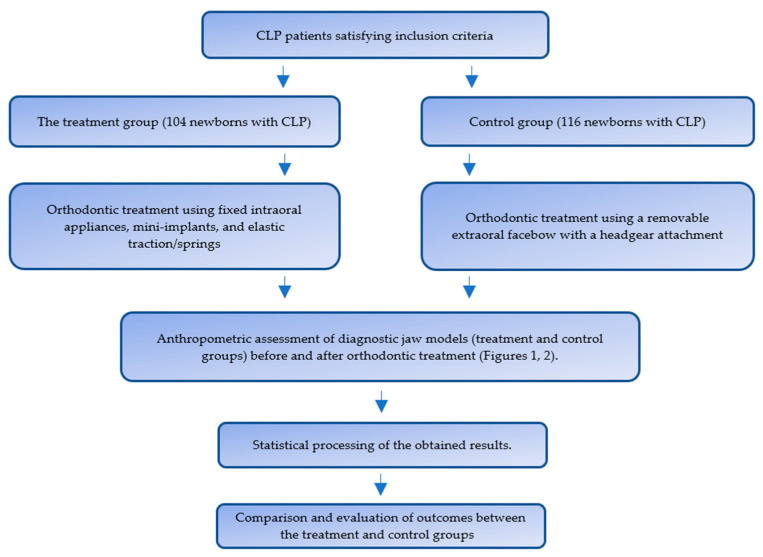
Study design.

All patients received diagnosis and treatment strictly in accordance with the study design, as outlined in Scheme 1.

The sample size was calculated using Cochran’s formula, n0 = (Z^2^ × *p* × (1 – *p*))/e^2^, where n0—the required sample size for an infinite population; Z—the Z-score corresponding to the desired confidence level (a quantile of the standard normal distribution); *p*—the estimated proportion of the characteristic in the population; and e—the margin of error. The calculation yielded a sample size that met the study requirements, with a 95% confidence level and a margin of error of ± 5%.

Group allocation was based on the following inclusion criteria: newborns aged up to 28 days with unilateral or bilateral cleft lip and palate (complete or partial), major congenital anomalies or systemic conditions that could interfere with the results. We excluded patients older than 28 days and those with systemic conditions that might affect rehabilitation outcomes. Patients were withdrawn if their parents refused to continue or did not comply with the prescribed treatment and the physician’s recommendations.

The study used a combination of clinical history and examination, along with imaging, photographic, anthropometric, and statistical methods, applied throughout the stages of comprehensive rehabilitation for children with CLP.

**Figure 1:**
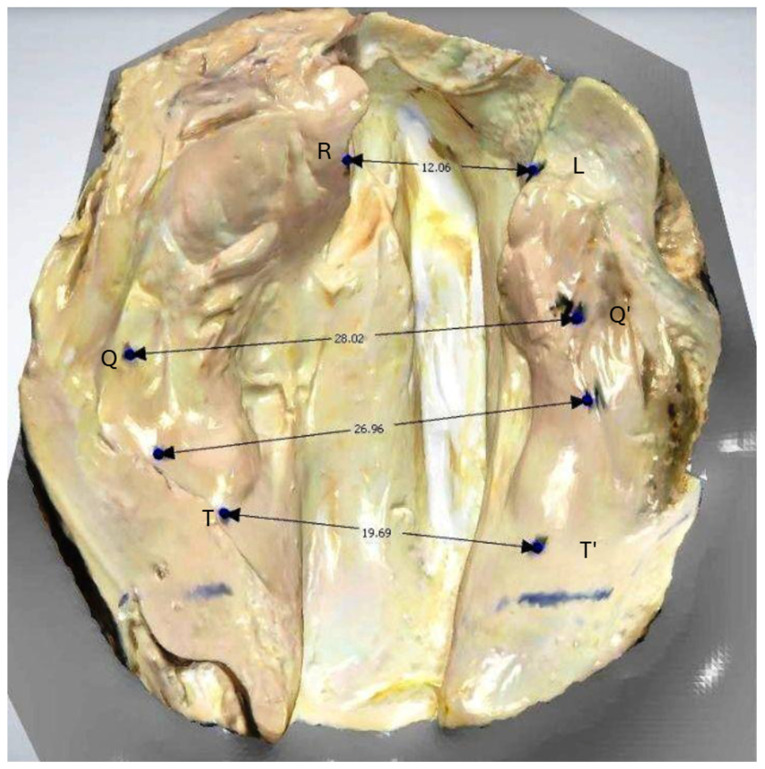
Key reference points on the graphical diagnostic model for geometric analysis of alveolar process fragment positioning in patients with unilateral cleft lip and palate. Notes: R—the anterior point of the right alveolar process fragment; L—the anterior point of the left alveolar process fragment; Q—the midpoint of the right alveolar process fragment length; Q’—the midpoint of the left alveolar process fragment length; T—the posterior point of the right alveolar process fragment; T’– the posterior point of the left alveolar process fragment; R-L—the distance between the anterior points of the alveolar process fragments; Q-Q’—the distance between the midpoints of the alveolar process fragments; T-T’—the distance between the posterior points of the alveolar process fragments.

**Figure 2:**
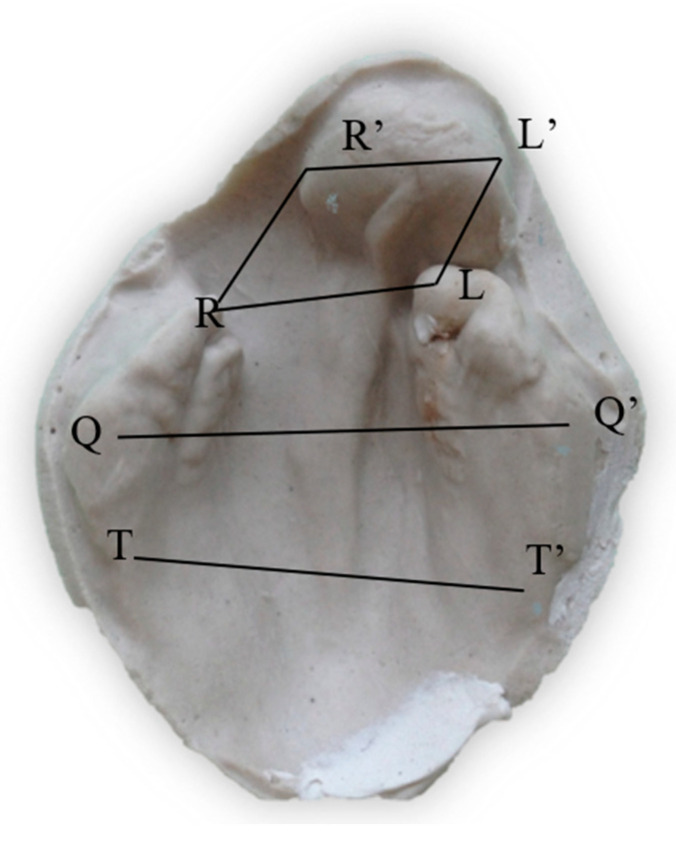
Key reference points on the diagnostic model for geometric analysis of the position of alveolar process fragments and premaxilla in patients with bilateral cleft lip and palate. Notes: R’—the anterior point of the intermaxillary bone on the right side; L’—the anterior point of the intermaxillary bone on the left side; R—the anterior point of the right alveolar process fragment; L—the anterior point of the left alveolar process fragment; Q—the midpoint of the right alveolar process fragment length; Q’—the midpoint of the left alveolar process fragment length; T—the posterior point of the right alveolar process fragment; T’—the posterior point of the left alveolar process fragment; R’-L’—the width of the intermaxillary bone; R-R’—the distance between the anterior point of the right alveolar process fragment and the intermaxillary bone; L-L’—the distance between the anterior point of the left alveolar process fragment and the intermaxillary bone; R-L—the distance between the anterior points of the alveolar process fragments; Q-Q’—the distance between the midpoints of the alveolar process fragments; T-T’—the distance between the posterior points of the alveolar process fragments.

Diagnosis and treatment were carried out in 104 newborns. The cohort included 48 infants with UCLP and 56 with BCLP, forming the treatment group. For every patient, anthropometric assessment of diagnostic jaw models was completed at baseline and upon treatment completion. As part of our routine assessment, we took anthropometric measurements of diagnostic jaw models from all patients before treatment and again at the end of treatment to track changes in the dental arches. The models were then photographed in a standard orientation and the images were uploaded to AUTOCAD 2026 software. With this software, we obtained the graphical shape of the alveolar ridges of the lateral fragments, the intermaxillary bone, and the vomer. We then marked reference points and took linear and angular measurements between them (Figure 1 and Figure 2). All data were recorded in custom tables and analysed statistically.

We performed radiographic examinations on the newborns before and after treatment, which allowed us to determine the severity of the diastema and the position of the alveolar fragments and the intermaxillary bone. For more severe deformities, or to locate tooth germs, we used cone-beam computed tomography (CBCT). This method gave clear images of the maxillofacial anatomy and helped us find the best site for mini-implant fixation.

**Figure 3:**
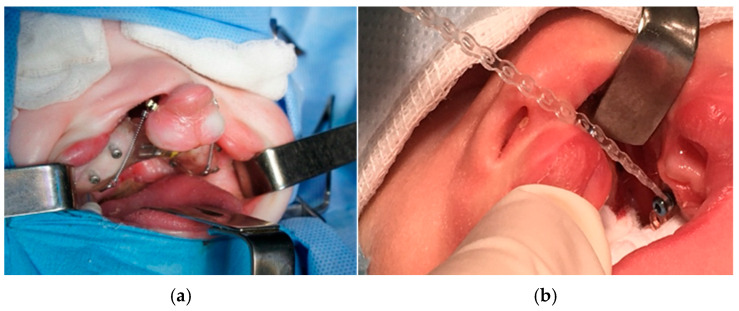
Fixation of the orthodontic appliance: patient with BCLP (**a**) and UCLP (**b**).

After the initial assessment, all 104 newborns with UCLP and BCLP required primary cheiloplasty after preliminary orthodontic preparation of the alveolar fragments and the intermaxillary bone. All patients in the treatment groups (n = 104) underwent orthodontic correction to reposition the alveolar fragments (in UCLP) and the intermaxillary bone (in BCLP) using a modified Latham appliance and a technique using mini-implants and elastic traction devices/springs (Figure 3).

Traction between the mini-implants was achieved using either an elastic chain connector or compression springs. The elastic chain underwent activation every three days (one link at a time, delivering 50 g of force); spring activation was verified with a dynamometer.

The active phase of orthodontic treatment lasted 20–25 days. After that, the elastic chain or springs were left in place without activation for a retention period of 20–30 days.

As a control group, we conducted a comparative analysis using retrospective data. We retrieved 116 historical medical records and corresponding control jaw models from our institutional archive for newborns diagnosed with UCLP and BCLP. These cases had undergone orthodontic correction of alveolar and premaxillary fragments using standard removable extraoral orthodontic appliances (facial sling with headgear) and served as comparison groups. Data were collected at baseline and after intervention.

The data were analysed using descriptive statistics. The frequency distribution of each variable was described using the following metrics:

(1) Central tendency measures reflected by the arithmetic mean value (ME or mean).
(2) Dispersion measures quantified by standard deviation (±SD).
(3) Distribution asymmetry and peakedness captured by the skewness coefficient (A) and kurtosis coefficient (E), respectively.

The formula for the arithmetic mean (ME) is expressed as

ME=(∑Mi)/n,

where ∑ denotes the sum, Mi represents the individual measurement values, and n is the total number of observations.

The formula for standard deviation (±SD) is given as

SD=√(∑(Mi−M)2)/n),

where ∑ denotes the sum, Mi represents an individual sample value, M is the mean value of the population, and n is the sample size.

We measured data on an interval scale and performed a preliminary exploratory analysis of distribution characteristics. Normality assessment followed a two-step approach: we used the Kolmogorov–Smirnov (K-S) test for samples larger than 50 observations, and the Shapiro–Wilk (S-W) test for smaller datasets (n < 50). In all cases, the distributions of the variables significantly departed from normal, so we rejected the normality hypothesis.

Therefore, we used non-parametric statistical tests to analyse within-group and between-group differences:

1. We used the Mann–Whitney U test for comparisons between independent groups;
2. We used the Wilcoxon signed-rank test for paired samples.

By ranking the absolute values of the measured variable, these methods reduce the effect of outliers and handle skewed data distributions.

The Mann–Whitney U test was calculated according to the following formula:

U=N1N2+(Nx(Nx+1)/2)−Tx,

where N denotes the total sample size, Nx is the size of the larger sample group, and T represents the greater of the two rank sums.

The Wilcoxon signed-rank test (T-Wilcoxon) was computed as

T = ∑Rr,

where ∑Rr denotes the sum of ranks corresponding to non-typical changes in the measured parameter.

To evaluate the new treatment, pairwise comparisons between groups were made to look for statistically significant differences in the parameters. Comparisons were made for within-group changes (in both control and experimental groups) before and after treatment, and for between-group differences at baseline and after treatment. Mean differences in parameter severity after treatment were also calculated.

The null hypothesis (H0) tested was that no differences existed between samples, i.e., the distributions were the same. The alternative hypothesis (H1) was that differences were present.

The significance level was set at *p* < 0.05 corresponding to a 95% confidence interval. When *p* < 0.05, H0 was rejected in favour of H1; when *p* > 0.05, H0 was accepted and H1 rejected. Statistically significant differences in mean values were considered present when the t-statistic was ≥2 (*p* < 0.05), and these were marked in the tables.

## 3. Results

Clinical examination and anthropometric analysis of diagnostic models revealed alveolar process deformation and a diastema between alveolar fragments in all infants with UCLP, specifically measured as the distance between the anterior points of the alveolar segments (R-L) and was 12.00 mm ± 0.67; between the midpoints (Q-Q’) 25.98 mm ± 0.69; and between the posterior points (T-T’) 19.00 mm ± 0.70. Oronasal communication was visible.

On oral examination of neonates with BCLP, deformation of the lateral alveolar fragments and a diastema were noted: the distance between the anterior point of the right alveolar process fragment and the intermaxillary bone (R-R’) was 14.00 mm ± 0.71; between the anterior point of the left alveolar fragment and the intermaxillary bone (L-L’) was 10.00 mm ± 0.67; and the distance between the anterior points of the right and left alveolar fragments (R-L) was 19.00 mm ± 0.70. Oronasal communication was visible.

**Figure 4:**
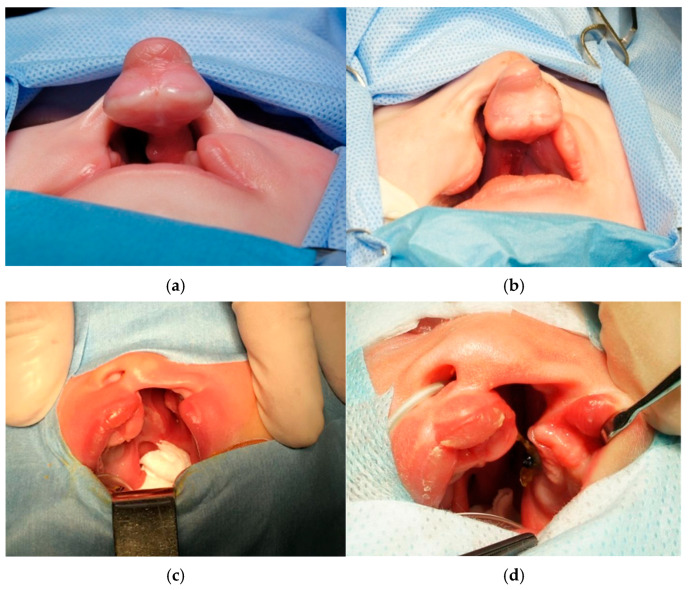
Patients in the treatment group with BCLP before (**a**) and after (**b**), and with UCLP before (**c**) and after (**d**), following correction of the alveolar process and intermaxillary bone using fixed orthodontic appliances.

**Figure 5:**
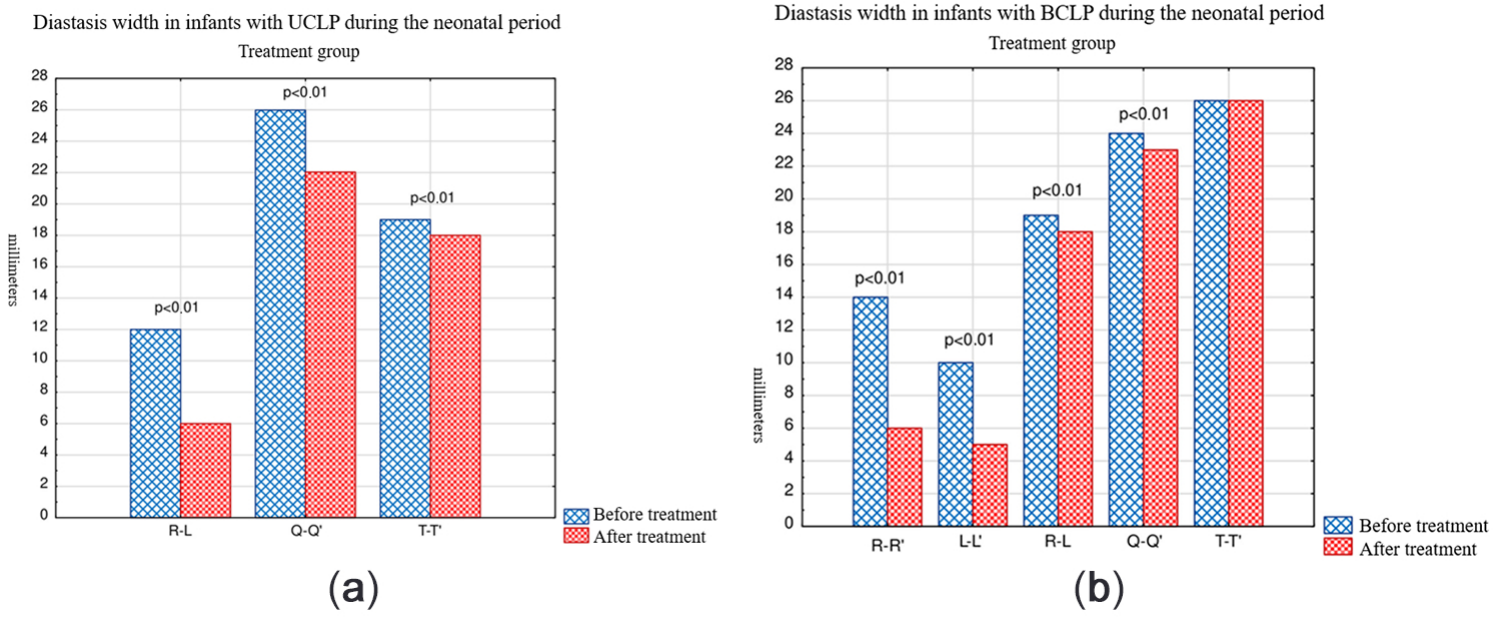
Changes in diastema in the treatment group: children with unilateral cleft lip and palate (**a**) and bilateral cleft lip and palate (**b**), before and after treatment.

Clinical and statistical data demonstrated that patients in the treatment groups achieved a reduction in the diastema between alveolar process fragments (UCLP) and the intermaxillary bone (BCLP) following orthodontic correction using a fixed intraoral orthodontic appliance, mini-implants, and springs/elastic traction, as confirmed by statistical analysis of the results (Figure 4 and Figure 5).

In neonates with UCLP, significant differences were detected across all diastema dimensions before and after orthodontic correction of the alveolar fragments (*p* < 0.01). After treatment, the distance between the anterior alveolar fragment points showed a marked reduction (R-L was 12.00 ± 0.67 mm before the correction compared to 6.00 ± 0.69 mm after the correction; *p*-value was 0.0000000016), while only slight changes were noted in the distances between midpoints (Q-Q’ was 25.98 ± 0.70 mm before the treatment versus 22.03 ± 0.68 mm after the treatment with the *p*-value 0.0000000016) and posterior points (T-T’ was 19.00 ± 0.68 mm before orthodontic correction of the alveolar fragments compared to 18.00 ± 0.71 mm after the orthodontic correction, *p*-value was 0.00000018) of the alveolar fragments. These results confirm the efficacy of orthodontic correction of alveolar fragments using mini-implants and springs/elastic traction in neonates with UCLP.

It should be emphasised that in newborns with BCLP from the treatment group, the diastema size after orthodontic correction of alveolar process fragment positioning and intermaxillary bone was significantly smaller compared to pre-treatment values across all assessed parameters—except for the distance between the posterior points of the alveolar process fragments (T-T’ was 26.00 ± 0.72 mm before the treatment compared to 26.00 ± 0.71 mm after the treatment; *p*-value was 0.976), which remained unchanged. This outcome is considered positive, as it confirms successful preservation of the maxillary width due to the inclusion of a widening screw in the orthodontic appliance design.

Statistical analysis showed that orthodontic correction of the alveolar fragments and intermaxillary bone using mini-implants combined with springs or elastic chains normalises the position of the intermaxillary bone and the shape of the maxilla. This approach facilitates primary cheilorhinoplasty and subsequent palatoplasty, shortens rehabilitation time, and leads to stable functional and aesthetic outcomes.

It should also be noted that we successfully preserved the distance between the anterior points of the alveolar process fragments (R-L was 18.00 ± 0.66 mm) following correction. This allowed proper positioning of the intermaxillary bone (R’-L’ was 16 mm) between the alveolar process fragments. The additional 2 mm space provided room for growth and development of the maxilla.

Thus, statistical data demonstrate that correction of the alveolar process fragment and intermaxillary bone positioning using a fixed orthodontic appliance in combination with mini-implants and springs/elastic traction enables normalisation of intermaxillary bone position and maxillary shape. This approach facilitates subsequent primary cheilorhinoplasty and, later, uranoplasty, while also reducing patient rehabilitation time for this condition—ultimately achieving stable aesthetic and functional outcomes.

Comparison of diastema dimensions between the treatment and control groups of newborns with UCLP before the start of treatment revealed statistically significant differences across all parameters (R-L was 12.00 ± 0.67 mm in the treatment group compared to 10.00 ± 0.70 mm in the control group; *p* = 0.00000000001; Q-Q’ was 25.98 ± 0.69 mm in the treatment group versus 24.00 ± 0.71 mm in the control group; *p* = 0.00000000001; T-T’ was 19.00 ± 0.70 mm in the treatment group compared to 17.00 ± 0.68 mm in the control group with a statistically significant difference (*p* = 0.00000000001)). The diastema measurements in the treatment group were significantly larger than those in the control group, indicating a more pronounced cleft severity in the treatment group of children with UCLP.

Further statistical analysis showed significant differences between the treatment group and the archival (control) group in children with bilateral cleft lip and palate for the following parameters: distance between the anterior point of the right alveolar fragment and the intermaxillary bone (R-R’ was 14.00 ± 0.71 mm in the treatment group compared to 13.00 ± 0.72 mm in the control group; *p* = 0.000001), distance between the anterior points of the alveolar fragments (R-L was 19.00 ± 0.70 mm in the treatment group versus 20.00 ± 0.73 mm in the control group; *p* = 0.000001), and distance between the posterior points of the alveolar fragments (T-T’ was 26.00 ± 0.68 mm in the treatment group compared to 27.00 ± 0.70 mm in the control group with a statistically significant difference (*p* = 0.000001)).

Thus, the pre-treatment data showed homogeneity between the compared groups and more severe deformity in the treatment groups.

**Figure 6:**
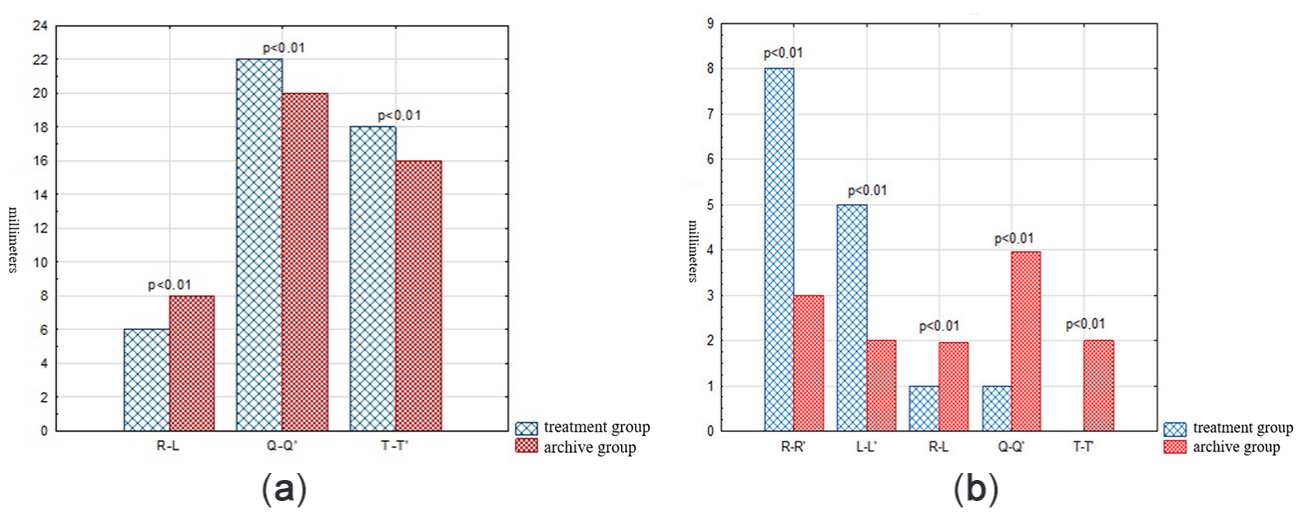
The severity of diastema in neonates with unilateral (**a**) and bilateral (**b**) cleft lip and palate after treatment in the treatment and control groups.

Post-treatment comparison of diagnostic data in neonatal UCLP and BCLP patients showed statistically significant differences in all diastema parameters between the treatment and control groups (Figure 6).

However, it is noteworthy that in patients with unilateral cleft lip and palate (UCLP), the distances between the midline points of the alveolar process fragments (Q-Q’ was 22.03 ± 0.68 in the treatment group compared to 20.00 ± 0.70 in the control group, *p* = 0.000000000008) and between the posterior points of the fragments (T-T’ was 18.00 ± 0.69 in the treatment group versus 16.00 ± 0.72 in the control group with a statistically significant difference, *p* = 0.00000000001) were more pronounced in the treatment group compared to the control group. At the same time, the distance between the anterior points of the alveolar process fragments (R-L) was significantly higher in the treatment group compared to the control group—6.00 ± 0.69 mm versus 8.00 ± 0.71 mm, respectively (*p* = 0.00000000001)—confirming the effectiveness of orthodontic correction in the treatment group.

Comparative analysis of the diastema in the treatment and control groups of newborns with bilateral cleft lip and palate after treatment revealed significant differences in all parameters except for the distance between the anterior points of the alveolar process fragments.

In the group of children who received orthodontic treatment using fixed intraoral appliances, mini-implants, and springs/elastic forces, the distance between the anterior point of the right alveolar fragment and the intermaxillary bone (R-R’) was 6.00 ± 0.69 mm in the treatment group, compared to 10.00 ± 0.72 mm in the control group (*p* = 0.000000001). Similarly, the distance between the anterior point of the left alveolar fragment and the intermaxillary bone (L-L’) was lower in the treatment group (5.00 ± 0.70 mm) than in the control group (8.00 ± 0.71 mm), with a statistically significant difference (*p* = 0.000000001). At the same time, parameters reflecting the distance between midline points of the alveolar fragments (Q-Q’) were higher in the treatment group (23.00 ± 0.71 mm) compared to the control group (20.00 ± 0.73 mm), reaching statistical significance (*p* = 0.000000001). Likewise, the distance between posterior points of the alveolar fragments (T-T’) also showed a higher value in the treatment cohort (26.00 ± 0.70 mm) versus the control cohort (25.00 ± 0.72 mm), and this difference was statistically significant (*p* = 0.0000006).

The findings demonstrate that, compared to removable appliances (control group), the correction with mini-implants and elastic traction or springs (treatment group) in infants with bilateral cleft lip and palate led to a greater reduction in the distance between the anterior point of the right alveolar fragment and the intermaxillary bone (∆R-R’ was 8 mm compared to 3 mm, *p* = 0.00000000002) and between the anterior point of the left alveolar fragment and the intermaxillary bone (∆L-L’ was 5 mm in the treatment group compared to 2 mm in the control group, *p* = 0.000000008). At the same time, this approach maintained the original dimensions between the anterior, mid, and posterior points of the alveolar segments, with only small changes observed (∆R-L = 1 mm, ∆Q-Q’ = 1 mm; ∆T-T’ = 0 mm).

In the control group, where removable appliances were used, there was a statistically significant narrowing between the anterior points of the alveolar fragments (R-L) in both UCLP and BCLP patients by the end of treatment (UCLP: 10.00 ± 0.71 mm before the treatment compared to 8.00 ± 0.71 mm after the treatment, *p*-value 0.000012; BCLP: R-L was 20.00 ± 0.72 mm before the treatment versus 18.03 ± 0.69 mm after the treatment with the *p*-value 0.000002), and between the posterior points (T-T’) of the alveolar fragments (UCLP: 17.00 ± 0.71 mm before the treatment compared to 16.00 ± 0.71 mm after the treatment with the *p*-value 0.000255; BCLP: T-T’ was 27.00 ± 0.72 mm before the treatment versus 25.00 ± 0.72 mm after the treatment, *p* = 0.000001). This narrowing may be unfavourable for the growth and development of the maxilla and, consequently, the mandible.

## 4. Discussion

The findings of our study demonstrate the high effectiveness of the proposed treatment approach for patients with cleft lip and palate, a conclusion supported by scientific evidence. The significance of reducing the diastasis between bone fragments—successfully achieved in our research—is consistent with the outcomes reported in large-scale multicentre studies. As noted by Ritesh Kalaskar et al. [19] and Badri Thiruvenkatachari [20], proper management of diastasis remains an important factor in successful treatment.

Quantitatively, our results strongly support the superiority of fixed intraoral appliances. We observed a markedly greater reduction in diastema compared to the control group: the treatment group achieved a ΔR-R′ reduction of 8 mm, whereas the control group saw only 3 mm (*p* = 0.00000000002), and a ΔL-L′ reduction of 5 mm compared with 2 mm in the control group (*p* = 0.000000008). Importantly, we maintained key dimensions with minimal change: ΔR-L = 1 mm, ΔQ-Q′ = 1 mm, and ΔT-T′ = 0 mm. These outcomes compare favourably with data from Kalaskar’s comparative trial [19], where the intraoral technique reduced diastema from 27.16 mm to 24.10 mm and the extraoral technique from 30.01 mm to 27.23 mm (both *p* < 0.01).

Of particular interest is the dynamic modelling of nasal cartilage in cases of complete unilateral clefts, described in Manu Prasad Shivanna’s study [11], which was also observed in our clinical practice. Our success in preserving the width of the upper jaw is further supported by studies investigating the influence of orthodontic appliances on jaw parameters. Justyna Pałka’s work [21] highlights the importance of maintaining transverse dimensions, demonstrating significantly greater improvements in both anterior width (reaching 1.38 M as opposed to 0.45 M in the comparison group, *p* < 0.001) and posterior width (0.93 M compared with 0.48 M, *p* < 0.001).

Gauri V. Patil’s study [22] emphasises the need to monitor maxillary width during orthodontic treatment, noting that controlled expansion reinforces dento skeletal structures. Patil details specific effects on the midpalatal suture, reporting anterior expansion of 2.42–4.0 mm and posterior expansion of 0.84–2.88 mm, stable over 3–12 months. This expansion also influences broader craniofacial parameters, including the mandibular plane angle, condylar processes remodelling, and nasal cavity width—the latter showing an average increase of 1.9 mm, with potential for up to 8–10 mm.

The preoperative preparation for cheilorhinoplasty implemented in our study corresponds with current approaches to presurgical management. Long-term outcomes described by Yoshitsugu Hattori [6] highlight the importance of careful preoperative planning, showing that patients undergo, on average, 5.9 ± 1.8 operations overall, with 2.2 ± 1.2 during the growth period and 1.6 ± 1.0 after skeletal maturation; notably, 7.5% of operations involve multiple procedures. This point is also supported by Manu Prasad Shivanna’s research [11], which emphasises the value of a comprehensive approach to surgical preparation. Importantly, our method eliminates the need for resections and osteotomies, thereby reducing the number of surgeries and facilitating the restoration of anatomical structures.

The reduced rehabilitation period observed in our study—a 50% reduction in preoperative orthodontic treatment duration—reflects broader trends described in the recent literature. The multidisciplinary approach outlined by Valeria Luzzi [5] offers an effective way to optimise patient recovery, while Shabnam Ajami’s work [12] shows that early intervention can significantly accelerate rehabilitation. Moreover, early rehabilitation interventions in infants play a critical role in restoring normal breathing, swallowing, and sucking functions which are essential for ensuring proper growth and development of the maxillofacial region [23,24,25,26,27], ultimately reducing the likelihood and extent of future invasive medical procedures. The stable aesthetic and functional outcomes we achieved are consistent with long-term follow-up data. Yoshitsugu Hattori’s research [6] demonstrates the long-term benefits of care for patients with bilateral clefts, and Arezoo Jahanbin’s study [28] on bone fixation techniques further supports our findings regarding result stability.

Quality-of-life metrics further validate our approach. ECOHIS scores revealed a clear pattern: the non-team management group reported higher scores, while the multidisciplinary team group showed measurable improvements (*p* < 0.001). This underscores the tangible impact of coordinated care.

Looking ahead, our findings open new possibilities for refining the treatment protocol. Key priorities include improving mini-implant placement protocols to minimise rejection rates, developing new fixed appliance designs with improved biomechanical properties, integrating digital technologies for more accurate treatment planning, and expanding the methodology’s applicability to a wider patient population.

At the same time, it is important to acknowledge the study’s limitations. These include the potential for mini-implant rejection, which requires further investigation into factors affecting successful osseointegration; the need to improve placement techniques to minimise tissue damage; and the requirement for longer follow-up periods to better assess term treatment outcomes.

Additional aspects of our research are also supported by the current literature. Daiana Opriş’s work [16] on patient quality of life, Nicola M Stock’s insights [17] into the psychosocial aspects of treatment, and Mark A Green’s analysis [4] of infant feeding challenges all contribute to our understanding of the proposed methodology’s overall effectiveness.

## 5. Conclusions

Comprehensive clinical and statistical assessment of the proposed orthodontic protocol reveals its substantial benefits in managing cleft lip and palate cases. In patients with unilateral cleft lip and palate (UCLP), application of a fixed appliance combined with mini-implants and elastic or spring forces successfully normalises the spatial relationship between maxillary alveolar fragments in 97.9% of instances, with a high statistical significance (*p* = 0.0000000016). Similarly, in bilateral cleft cases (BCLP), the same approach effectively eliminates protrusive positioning of the intermaxillary bone in 96.42% of patients, demonstrating even greater statistical significance (*p* = 0.00000000007).

These outcomes strongly support the clinical value of the technique. Furthermore, direct comparative analysis against traditional treatment using removable extraoral appliances reveals a statistically significant advantage in favour of the fixed intraoral system (*p* < 0.01), highlighting its potential as a superior alternative in early orthodontic management of cleft anomalies.

The observed 50% reduction in presurgical orthodontic treatment duration further supports the protocol’s efficiency, potentially enabling earlier primary cheilorhinoplasty and uranoplasty with stable anatomical and functional outcomes.

However, exploring the integration of digital technologies—such as 3D printing or AI-driven treatment planning—may further enhance precision and accessibility, making advanced CLP care more widely available.

In conclusion, the integrated preoperative orthodontic and surgical protocol shows strong potential to advance CLP management. Its successful global implementation depends on protocol standardisation, specialised training, and ongoing refinement guided by future evidence.

## Data Availability

The original contributions presented in this study are included in the article. Further inquiries can be directed to the corresponding author.

## Abbreviations

The following abbreviations are used in this manuscript:

UCLP	Unilateral cleft lip and palate
BCLP	Bilateral cleft lip and palate
CLP	Cleft lip and palate
CBCT	Cone-beam computed tomography
n	Number
Me ± SD	Median ± standard deviation
*p*	*p*-value
OR	Odds ratio
FL	Federal Law
